# Point prevalence of antibiotic usage in major referral hospital in Turkey

**DOI:** 10.1371/journal.pone.0296900

**Published:** 2024-01-31

**Authors:** Müge Ayhan, Belgin Coşkun, Bircan Kayaaslan, İmran Hasanoğlu, Ayşe Kaya Kalem, Fatma Eser, Yeşim Aybar Bilir, Serpil Ünlü, Rahmet Güner

**Affiliations:** 1 Department of Infectious Diseases and Clinical Microbiology, Ankara City Hospital, Ankara, Turkey; 2 Department of Infectious Diseases and Clinical Microbiology, Ankara Yıldırım Beyazıt University, Ankara City Hospital, Ankara, Turkey; Shiraz University of Medical Sciences, ISLAMIC REPUBLIC OF IRAN

## Abstract

**Introduction:**

The most important and undesirable consequence of inappropriate antibiotic use is the spread of antibiotic resistance, increased adverse effects, increased mortality and healthcare costs. We aimed to assess antibiotic usage characteristics in inpatient setting in our center.

**Materials and methods:**

A one-day, single center point-prevalence study was carried out on June 9th 2021, in Ankara City Hospital in Turkey. Data of antibiotic consumption, appropriateness of usage and predictors of inappropriate use in adult patients were evaluated.

**Results:**

Out of 2640 adult patients, 893 (33.8%) were receiving at least one antibiotic. A total of 1212 antibiotic prescriptions with an average of 1.44±0.64 were found. Antibiotics were most commonly used for therapeutic purpose (84.7%), followed by surgical prophylaxis (11.6%). Majority of therapeutically used antibiotics were empirical (67.9%). Infectious diseases consultation was present in 68.3% with a compliance rate of 95.7%. Rate of inappropriate use was 20%. The most frequent cause of inappropriateness was unnecessary use (52.5%). Most commonly and most inappropriately used antibiotics were carbapenems (17.5%) and first generation cephalosporins (38.7%), respectively. Most of the inappropriateness observed in first-generation cephalosporins was due to inappropriate longer surgical prophylaxis. While age is an independent risk factor for inappropriate antibiotic use (p = 0.042), COVID-19 unit admission, use for therapeutic purpose and infectious diseases consultation were protective factors (p<0.001, p<0.001, p<0.001).

**Conclusion:**

Rate of inappropriate use was low, but inappropriate surgical prophylaxis remains an important problem in surgical units. There is a considerable need to implement an antimicrobial stewardship program that focuses on surgical prophylaxis practices.

## Introduction

Infectious diseases had been the most common cause of death in humans until antibiotics were used as drugs and introduced as “the magic bullet”. However, as a result of their widespread and excessive use, a new era has been encountered in which clinicians struggle with drug-resistant pathogens [1, 2]. With the increasing frequency of resistance and the need to reverse resistance patterns, worldwide calls were made by health authorities for controlled and rational antibiotic prescribing for inpatients and outpatients through the implementation of antibiotic stewardship programmes [3, 4].

Inappropriate antibiotic use is associated with increased hospital stay, healthcare costs. It also causes increased antibiotic resistance and leads to adverse patient outcomes such as increased morbidity and mortality as it limits available treatment options [3, 5]. As seen all over the world, antimicrobial resistance in Turkey has reached an alarming level. While carbapenem resistance is observed at a rate of up to 90% in *A*.*baumannii* strains, it is observed over 50% in *Klebsiella spp*. [6]. The rate and availability of antibiotic use are closely related to the development of resistance [7–9]. In our country, antibiotics are the most frequent used drugs and constitute approximately 20% of the drug market, despite cardiovascular drugs are the most frequently used drugs worldwide. Unfortunately, unnecessary and irrational use is also frequently observed [10, 11].

As a result of increasing antimicrobial resistance rates globally, a need for broader-spectrum and greater use of antibiotics has developed. Some antibiotics which are kept for special occasions or that were in use in the past had to be reused [12]. As a result, the World Health Organization announced a global action plan on antimicrobial resistance at the 68th World Health Assembly in 2015 [13]. Main goal of this action plan is to reduce inappropriate antibiotic use and associated antimicrobial resistance. To achieve this goal and to identify areas that can be improved, surveillance of antimicrobial use is very important. Point prevalence surveys are useful tools to collect data about antimicrobial use and to determine units requiring corrective measures. In addition, repeated surveys are also useful in order to evaluate the effects of the corrections made in the problematic areas [14, 15] In this study, we aimed to determine the antibiotic usage characteristics, rate and causes of inappropriate antibiotic use, most prevalent used antibiotic classes and risk factors for inappropriate use in hospitalized patients in the major referral hospital in our country.

## Materials and methods

### Study design and setting

This study was performed as a one-center point prevalence study on June 9th 2021 that focused on determining the antibiotic consumption and appropriateness of usage among adult inpatients in Ankara City Hospital, the main tertiary healthcare facility of Ankara, the capital of Turkey.

All adult patients in six buildings (general, cardiovascular surgery, neurology-orthopaedics, obstetrics and gynecology, oncology and physical therapy and rehabilitation) of the hospital were visited by infection control teams (a total of twenty two infection control nurses) and three infection control doctors, and data of patients who received at least one systemic antibiotic (parenteral of orally) were recorded. Pediatric units and emergency department were excluded.

### Data collection

Total bed capacity and bed occupancy rate on the day of survey was obtained from hospital statistics unit. Antibiotic usage data was collected by using a standardized form which was prepared by two of the researchers (MA and BC). In this form, hospital data, patient’s age, in which clinic patient is hospitalized, infection site, if any, the antibiotics used, the dose and duration of the antibiotic, the indication for antibiotic (therapeutic, medical prophylaxis or surgical prophylaxis), whether there is an infectious diseases consultation, if any, whether there is compliance with the consultation, were recorded.

Since COVID-19 patients were followed in separate units from other patients, the status of being followed in COVID-19 units was also recorded. If there were signs, symptoms and laboratory findings of an other infection in addition to the findings of main disease in COVID-19 patients, this infection site was recorded. In addition, if there were findings suggestive of lower respiratory tract infection in addition to COVID-19 involvement, it was recorded as pneumonia.

Topical, inhaler, intra-peritoneal, antiviral, antifungal, antiparasitic and antituberculosis treatments were excluded. All systemic antibiotics (parenteral and orally used) were recorded.

Indication for antibiotic was classified as surgical prophylaxis, medical prophylaxis or therapeutic purpose. Treatment types were classified as empirical or definitive. Empirical treatment was defined as initial antibiotic treatment if there is a possible infection, which is directed against an anticipated and likely cause of suspected infection, when there is no culture taken or culture and susceptibility results are pending. Definitive or targeted antibiotic therapy was defined as the administration of antibiotics that are found to be effective according to the culture and antibiotic susceptibility results.

Medical prophylaxis was defined as administration of antibiotics given to prevent bacterial infections in susceptible patient groups (eg. Patients receiving antibiotic prophylaxis during hematopoetic stem cell transplantation, patients receiving meningitis prophylaxis for possible cerebrospinal fluid leakage, pregnant women with suspected/susceptible to preterm/premature rupture of membranes to prevent early-onset neonatal Group B streptococcal disease, patients with cirrhosis and receiving prophylactic antibiotics to prevent spontaneous bacterial peritonitis and to prevent infective endocarditis in high risk patients).

Surgical antibiotic prophylaxis was defined as as the prevention of infectious complications by administering an effective antimicrobial agent prior to exposure to possible contamination during surgery. Determination of appropriateness of a prescribed antibiotic was based on standard international treatment guidelines or principles for diagnosis and treatment of infectious diseases [16–24]. Standard recommendations for surgical prophylaxis were used. [25, 26]. Antibiotic was defined as inappropriate if 1) there is unnecessary use of antibiotics without an indication, 2) the prescribed antibiotic is used at a wrong or inadequate dose, 3) the length of therapy is longer than the recommended duration for the indication, 4) the causative agent is established and there is resistance to the prescribed antibiotic, or it is not suitable for the infection site. Existence of infectious diseases consultation was recorded and compliance with the recommendations for antibiotic use in these consultations was evaluated. All forms were reviewed by two of the researchers who did not participate in the data collection (MA and BC) and appropriateness and causes of inappropriate antibiotic use were evaluated. Predictors of inappropriate antibiotic use were also evaluated.

### Statistical analysis

Statistical analysis was performed using Statistical Package for the Social Sciences (SPSS) version 25. Categorical data was reported as frequency with percentage while continuous data was presented as mean ± standard deviation. Categorical data was compared using Chi-square test between medical and surgical units. Univariable and multivariable analysis were used to determine predictors of inappropriate antibiotic use. Univariable factors were screened and thoe with p values below 0.2 were included in a backward elimination and foorward stepwise multivariable model. Statistical significance was as p<0.05 (using the Wald test). Odds ratios, 95% confidence intervals (CI) were calculated for each factor in the final model.

### Ethics statement

The authors declare that the procedures were followed according to the regulations established by the Clinical Research and Ethics Committee and to the Helsinki Declaration of the World Medical Association updated in 2013. This study was approved by Ethics Commitee of Ankara City Hospital No:1 (Approval number: E1-21-1748). Need for informed consent was waived by the ethical committee.

## Results

Adult bed capacity on the study day was 3390 and there were 2640 hospitalized patients (rate of occupied adult beds was 77.9%). While adult intensive care bed capacity was 966, the intensive care bed occupancy rate was 72.8%. One thousand seven hundred and thirty-four patients were hospitalized in medical units, while 906 patients were hospitalized in surgical units.

A total of 893 (33.8%) patients were receiving at least one antibiotic, 498 (55.8%) of them were male, sex distribution were similar between medical and surgical units (%55.4 and %56, respectively) The mean age of study population was 59.63±19.24 years. Five hundred eighty one (65.1%) patients were in wards and 312 (34.9%) patients were in intensive care units (ICU). Most common infection site was lower repiratory tract (26.2%), which was followed by urinary tract (17%) and intraabdominal site (15%) (Table 1). Of the patients, a total of 146 (16.3%) were hospitalized for COVID-19 infection.

**Table 1:** Patients’ characteristics included to the study.

	Total (n = 893)
**Age, *mean±SD*** *	59.6 (±19.2)
**Male sex, n (%)**	498 (55.8)
**Infection site, n (%)**	
Lower respiratory tract	234 (26.2)
Urinary tract	152 (17.0)
Intraabdominal site	134 (15.0)
Cardiovascular system	125 (13.9)
Skin&soft tissue	71 (7.9)
Surgical site	25 (2.8)
Central nervous system	13 (1.4)
Bone&joint	10 (1.1)
Genitourinary tract	10 (1.1)

*SD: Standard deviation

**ICU: Intensive care unit

Patients have had a total of 1212 antibiotic prescriptions with an average of 1.44±0.64 prescriptions per patient (Table 2). While 559 (62.6%) of the patients using antibiotics were in the medical units, 334 (37.4%) patients were in the surgical units.

**Table 2:** Distribution of antibiotics on the day of study, n (%).

**One**	571 (63.9)
**Two**	258 (28.9)
**Three**	59 (6.6)
**Four**	5 (0.6)
**Average number of antibiotics used (mean±SD** * **)**	1.4±0.6

*SD: Standard deviation

Antibiotics were used for therapeutic purpose (84.7%), and for prophylactic purpose (15.3). In 67.9% of the patients, antibiotics used for therapeutic purpose were empirical and in 32.1% were definitive. Infectious diseases consultation was present in 68.3% of the patients using antibiotics, and compliance with these consultations was observed in 95.7%. Overall, 20% of antibiotic use was inappropriate and the causes of inappropriateness were unnecessary use (52.5%), inappropriate dose (0.5%), inappropriate longer duration (30.1%) and incompatible use with infective focus or infective agent (16.7%).

While the use of antibiotics for therapeutic purpose was observed more frequently in medical units (539/559, 96.4%) than the surgical units (217/334, 64.9%) (p<0.001), prophylactic use was observed more frequently in surgical units (117/334, 35.0%) than the medical units (20/559, 3.5%) (p<0.001).

The presence of infectious diseases consultation was observed more frequently in medical units (82.1%, p<0.001), the rate of compliance with infectious diseases consultation was both high in medical and surgical units (96% ve 94% respectively, p = 0.234). Inappropriate antibiotic use was significantly higher in surgical units than in medical units (25.4% and 16.8% respectively, p<0.001).

The most common cause of inappropriate antibiotic use was unnecessary use and inappropriate longer duration than recommended in medical units, while unnecessary use was in surgical units. Unnecessary use was more frequent in surgical units than in medical units (65.8%, P<0.001), inappropriate longer duration was observed more frequently in medical units than in surgical units (40.4%, P<0.001) (Table 3).

**Table 3:** Comparison of antibiotic usage characteristics between medical and surgical units.

	Total (n = 893)	Medical unit (n = 559)	Surgical unit (n = 334)	P value
**Indication, n (%)**				
Therapeutic	756 (84.7)	539 (96.4)	217 (64.9)	***<0*.*001***
Prophylaxis	137 (15.3)	20 (3.5)	117 (35.0)	***<0*.*001***
**Treatment type for therapeutic purpose, n (%)**				
Empirical	514 (67.9)	357 (66.2)	157 (72.3)	***<0*.*001***
Definitive	242 (32.1)	182 (33.8)	60 (27.6)	***<0*.*001***
**Infectious disease consultation, n (%)**	610 (68.3)	459 (82.1)	151 (45.2)	***<0*.*001***
Compliance with consultation	584 (95.7)	442 (96)	142 (94)	0.234
**Inappropriate antibiotic use, n (%)**	179 (20)	94 (16.8)	85 (25.4)	***<0*.*001***
**Causes of inappropriateness, n (%)**				
Unnecessary use	94 (52.5)	38 (40.4)	56 (65.8)	***<0*.*001***
Inappropriate dose	1 (0.5)	1 (1.1)	0 (0)	0.222
Inappropriate longer duration	54 (30.1)	38 (40.4)	16 (18.8)	***<0*.*001***
Incompatible with focus or infectious agent	30 (16.7)	17 (18)	13 (15.3)	0.978

The most commonly used antibiotic class was carbapenems which were received by 213 patients (17.5%). It was followed by third generation cephalosporins and piperacillin-tazobactam which were received by 204 patients (16.8%) and 165 patients (13.6%), respectively (Table 4). Antibiotics which were most frequently used inappropriately on the survey day were the first generation cephalosporins (38.7%), sulbactam ampicillin (24.4%) and piperacillin tazobactam (20.6%).

**Table 4:** The inappropriateness of antibiotic prescriptions according to class of antibiotic.

Class of antibiotic	Total (n = 1212)	Proportion of inappropriateness, n(%)
**Penicillins, n (%)**		
Cristalized penicillins	13 (1)	-
Amoxicillin-clavulonate	12 (0.9)	1 (8.3)
Sulbactam-ampicillin	45 (3.7)	11 (24.4)
Piperacillin-tazobactam	165 (13.6)	34 (20.6)
**Cephalosporins, n (%)**		
1^st^ generation cephalosporins	80 (6.6)	31 (38.7)
3^rd^ generation cephalosporins	204 (16.8)	40 (19.6)
Cefepim	22 (1.8)	4 (18.2)
Cephoperazone-sulbactam	2 (0.1)	-
**Carbapenems, n (%)**	213 (17.5)	19 (8.9)
**Glycopeptides, n (%)**	150 (12.3)	29 (19.3)
**Daptomycin, n (%)**	11 (0.9)	1 (9)
**Linezolide, n (%)**	6 (0.5)	2 (33.3)
**Polymixins, n (%)**	79 (6.5)	15 (18.9)
**Quinolones, n (%)**	64 (5.2)	12 (18.7)
**Tygecycline, n (%)**	27 (2.2)	4 (14.8)
**Doxycycline, n (%)**	4 (0.3)	3 (75)
**Trimethoprim-sulfamethoxazole, n (%)**	22 (1.8)	3 (13.6)
**Macrolides, n (%)**	20 (1.7)	-
**Aminoglycosides, n (%)**	19 (1.5)	2 (10.5)
**Fosfomycin (iv form), n (%)**	4 (0.3)	1 (25)
**Metronidazole, n (%)**	49 (4)	3 (6.1)
**Clindamycin, n (%)**	3 (0.2)	-

In multivariable logistic regression analysis, age (OR 1.012, 95% CI 1.000–1.024), COVID-19 unit admission (OR 0.160, 95% CI 0.090–0.285), therapeutic purpose (OR 0.139, 95% CI 0.082–0.236) and presence of an infectious diseases consultation (OR 0.124, 95% CI 0.079–0.195) were independent predictors of inappropriate antibiotic use (Table 5).

**Table 5:** Risk factors for inappropriate antibiotic use.

	Univariable				Multivariable			
	OR	95% confidence interval		P value	OR	95% confidence interval		P value
		Lower bound	Upper bound			Lower bound	Upper bound	
**Age**	0.988	0.977	1.000	0.013	1.012	1.000	1.024	0.042
**Male**	0.685	0.666	1.056	0.087				
**COVID-19 unit admission**	0.590	1.185	10.938	<0.001	0.160	0.009	0.285	<0.001
**Type of department, surgery unit**	1.493	0.865	2.575	0.150				
**Type of unit, ward**	1.416	0.884	2.270	0.148				
**Indication, treatment**	0.631	0.143	0.954	<0.001	0.139	0.082	.0236	<0.001
**Prophylaxis (medical+surgical)**	1.291	0.506	3.298	0.593				
**Presence of infectious disease consultation**	0.118	0.006	0.212	<0.001	0.124	0.079	0.195	<0.001

## Discussion

In this point prevalence study, we aimed to examine prevalence of antibiotic use, the rate and the possible causes of inappropriate antibiotic use in the largest hospital of our country, to identify incorrect practices and to guide the improvements to be made for rational antibiotic use. In present study, 33.8% of hospitalized adult patients were using at least one antibiotic. In previous studies from our country, the antibiotic prescription was reported at different rates varying from 27.2% to 44.8% [15, 27–30]. In a study from Australia, a rate of 43% antibiotic use was reported, while in a multicenter study including 13 centers in China, this rate was reported as 56% [5, 14] In a study evaluating antibiotic use in 53 countries in Europe, rates of antibiotic use were reported as 15.6% in Northern Europe and 43.9% in Eastern Europe.

In Pakistan, Nigeria and Tanzania, higher rates of antibiotic use were reported ranging from 62% to 80.1% [31–33]. While the rate of antibiotic use in our study was similar to previous studies in our country [7, 28, 29], it was observed with a higher rate than some of the European countries, it is observed lower than in Asia and some European countries [14, 32, 34].

Average antibiotic prescription was 1.4±0.6 prescriptions per patient in our study and this was consistent with the data that has been reported in a multicenter recent point prevalence study from our country (average antibiotic prescription rate was 1.37±0.642 per patient) [7].

Of antibiotics, 84.7% were given for therapeutic indication, followed by surgical prophylaxis (11.6%). Approximately two thirds of all prescribed antibiotics (67.9%) were given empirically. Empirical antibiotic use has been reported in the literature at rates ranging from 49% to 96.2% [5, 27, 30, 32]. If the culture based definitive treatment rates can be increased and rates of empirical use can be reduced, early de-escalation can be implemented.

Although principles of rational and appropriate use of antimicrobials are well-known, inappropriate antibiotic use has been increasing. Increased healthcare costs, adverse reactions, emergence and spread of multi-drug resistant pathogens are the main problems resulting from the widespread excessive use of antibiotics [27].

The most effective and feasible way to control the spread of multi-drug resistant microorganisms is to provide rational antibiotic use [28, 32].

Inappropriate antibiotic use is a worldwide problem. In our study, the rate of inappropriate use was 20% and was quite lower than previous studies. In the previous studies, rates of inappropriateness varied between 25.8% and 54.3% from our country [28–30]. Ingram et al. reported 47% inappropriate antibiotic usage [5]. The reasons why the rate of inappropriate use is lower in our study were thought that patients were consulted with the infectious diseases department at a high rate, compliance with those consultations was high and both infectious diseases specialists and infection control committee teams visited every patient daily. Inappropriate antibiotic use was significantly higher in surgical units. This is because the majority of surgical prophylaxis are given longer than 24 hours. The problem of unnecessary longer duration of surgical prophylaxis has been reported in many previous studies before [35–37]. Current guidelines suggest single-dose surgical prophylaxis but acknowledge that if postoperative doses are still considered, then surgical antibiotic prophylaxis should not continue more than 24 hours [25, 26].

Unnecessary use and inappropriate longer duration were the most common causes of inappropriate use in our study. In previous studies, unjustified and unnecessary use, incorrect choice of antibiotics, incorrect dose and duration, treatment of colonizing and contamining agents were reported as common reasons for inappropriate antimicrobial use [5, 29, 38]. In our study, the rates of incorrect agent choice and incorrect dose were lower. In a study from Netherlands, in 37.4% of the patients, antimicrobial therapy was found to be inappropriate and in %14.9 antibiotic choice was incorrect for infective focus or agent [39] In the present study, carbapenems (17.5%) were the most common prescribed antibiotics and followed by third generation cephalosporins (16.8%) and piperacillin-tazobactam (13.6%). In previous studies third generation cephalosporins were the most frequent used antibiotic group [14, 31]. Carbapenem use was found to be quite high, but the inappropriate use was 8.9%. The high number of intensive care beds and the frequent occurrence of infections due to multi-resistant microorganisms were thought to be the reason for this high rate. In a previous study in our center, the rate of multi-drug resistant microorganisms was found to be 66% in patients who have pyelonephritis, while the rate of extended spectrum beta-lactamases was reported as 64.4% in *Enterobacterales* strains [40]. Carbapenems have an important place in the treatment of nosocomial infections [41]. If they continue to be used excessively, they will not be an available treatment option in the near future [7]. Antibiotic stewardship programmes should focus on carbapenem indications, alternative antibiotics and early treatment de-escalation [15, 42]. Almost all of the inappropriate use which was observed in first generation cephalosporins were because of unnecessarily prolonged surgical prophylaxis. Inappropriate and prolonged surgical antibiotic prophylaxis is associated with increased antimicrobial resistance [43], increased risk of *Clostridium difficile* infection, and increased antibiotic-related adverse events [36] Therefore, it is of great importance to increase compliance with the guidelines for surgical antibiotic prophylaxis.

Older age was significantly associated with inappropriate use. COVID-19 unit admission, use for therapeutic indication and presence of an infectious disease consultation were protective against inappropriate use. In a multicenter study, older age, prescription in summer were detected as risk factors for initial inappropriate antibiotic use, while end-stage renal disease was found to be a protective factor from inappropriate use. In the same study patients receiving cefepime or piperacillin-tazobactam were reported to be at greater risk of receiving inappropriate antibiotics on days 3–5 due to the failure to de-escalate [44]. Elderly people are often hospitalized as there is a higher risk of infection and infection-related adverse outcomes, multiple comorbidities and polypharmacotherapy. Signs and symptoms of infection may be atypical, and this may sometimes lead overdiagnose and inappropriate antibiotic use [45].

Although inappropriate use of antimicrobials in COVID-19 patients has been reported before [46, 47], in our study being in a COVID-19 unit was a protective factor from inappropriate antibiotic use. This can be explained by two reasons. First, it was thought that in our center, COVID-19 units have been visited by an infectious diseases specialist every day and patient charts and antibiotics are reviewed for appropriateness. The second possible cause was thought that our center have been the major COVID-19 center in our country. More severe patients have been followed. In some patients it is really hard to make a differential diagnosis between bacterial pneumonia and COVID-19 infection. Researchers may have been unintentionally biased.

Using antibiotics for therapeutic purpose was also a protective factor. Inappropriate antibiotic use was reported more frequently in patients using antibiotics for prophylaxis than in patients using antibiotics for therapeutic purpose [5]. This study is the first point prevalence study performed in the highest number of patients as a single center in our country. Point prevalence surveys are inexpensive, effortless and easily repeatable type of studies [29, 48]. It may not be appropriate to make a comparison between different centers as the characteristics of the centers may vary. However, the effectiveness of the improvements to be made in that center and the antibiotic stewardship program, if any, can be evaluated with periodic surveys in the same center. High number of patients, diversity of antibiotics due to being a large center and a detailed assessment of antibiotic use are strengths of our study. And there were several limitations. First, the relationship between antibiotic use and the cause of hospitalization, disease severity could not be evaluated. Secondly, the antibiotic usage preferences could not be detailed according to units. Our center is the largest center in the country where immunosuppressive patients, organ and tissue transplants are managed. Many patients take antiviral, antifungal drugs, etc. One last limitation was that the use of antimicrobials (antifungal, antiviral, antituberculosis, etc.) other than antibacterial drugs was excluded.

## Conclusion

In conclusion, prevalence of antibiotic consumption in our hospital was similar to the previous literature from our country, but it was higher than some European countries. Most of the antibiotic use was empirical. Rate of inappropriate use was lower than other studies, but inappropriate prolonged surgical prophylaxis remains a problem in surgical units. In each center, appropriate and rational antibiotic usage guidelines should be developed in accordance with the patient profiles and the capacity of the center, and compliance with these guidelines should be monitored periodically with point prevalence studies.
