# Correction: Point prevalence of antibiotic usage in major referral hospital in Turkey

**DOI:** 10.1371/journal.pone.0304142

**Published:** 2024-05-16

**Authors:** The PLOS ONE Editors

## Notice of republication

This article [1] was republished on February 16^th^, 2024, to address an issue identified post-publication. An updated version of S1 File is provided with this notice. Please download this article again to view the correct version.

The article’s Data Availability statement is updated to: "The primary data underlying this article are included within the article and Supporting Information files."

## Supporting information

S1 File Primary data (XLSX)

## References

[1] Ayhan M, Coşkun B, Kayaaslan B, Hasanoğlu İ, Kalem AK, Eser F, et al. (2024) Point prevalence of antibiotic usage in major referral hospital in Turkey. PLoS ONE 19(1): e0296900. doi: 10.1371/journal.pone.0296900 38295065 PMC10830045

